# Calcineurin Inhibitors in Atopic Dermatitis: Balancing Tradition with Emerging Therapeutics

**DOI:** 10.3390/medsci14020297

**Published:** 2026-06-08

**Authors:** Rakesh Kumar, Syed Arman Rabbani, Mohamed El-Tanani, Shrestha Sharma, Manita Saini

**Affiliations:** 1 Amity Institute of Pharmacy, Amity University, Gurgaon 122413, Haryana, India; rakeshk.bhardwaj98@gmail.com (R.K.); manitasaini44@gmail.com (M.S.); 2 Clinical Pharmacy & Pharmacology, Jagannath University, Bahadurgarh 124507, Haryana, India; 3 RAK College of Pharmacy, RAK Medical and Health Sciences University, Ras Al Khaimah 11172, United Arab Emirates; 4 Geeta Institute of Pharmacy, Geeta University, Panipat 132145, Haryana, India

**Keywords:** atopic dermatitis, calcineurin inhibitors, tacrolimus, pimecrolimus, topical immunomodulators, nanocarriers, steroid-sparing therapy, T-cell signaling, inflammatory skin disease

## Abstract

Atopic dermatitis (AD) is a common chronic inflammatory condition of the skin that has increased dramatically over the past decade and significantly impacts individual quality of life. Corticosteroids are still the primary therapy for AD, but there are limitations to their continued use due to potential adverse effects, particularly when used in sensitive areas. Topical calcineurin inhibitors (CNIs), such as tacrolimus and pimecrolimus, are available as a safe, steroid-sparing alternative that directly inhibit calcineurin-mediated activation of T cells and have been shown to be efficacious according to varying clinical study designs including randomized controlled trials, registry studies and meta-analyses. Although there was controversy regarding the safety of CNIs subsequent to the FDA’s black-box warning in 2006, the preponderance of evidence supports their continued safety when used as directed. In contrast to biologics and JAK inhibitors, CNIs occupy an inherently unique therapeutic niche for use in pediatric patients, have demonstrated historical efficacy, and can provide localized affordable treatment in sensitive areas including the face, eyelids and intertriginous surfaces. Furthermore, the role of CNIs in the context of precision dermatology continues to be defined through new innovations including barrier-repair strategies used in combination with topical medications, microneedle systems, and nanocarrier formulations. Hence, the role of CNIs in the current AD treatment paradigm is crucial and lies at the interface between topical corticosteroids and systemic immunomodulatory agents. The narrative review discusses recent advances in formulation strategies, combination approaches, and targeted delivery systems, underscoring how CNIs continue to bridge established practice and emerging therapeutic innovation in AD.

## 1. Introduction

**Figure 1:**
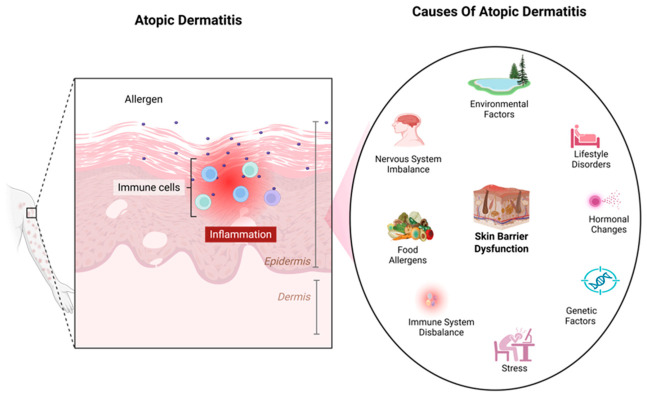
**Pathophysiology of skin barrier dysfunction and inflammation.** Epidermal barrier dysfunction permits allergen and microbial penetration, leading to activation of antigen-presenting cells (Langerhans cells and dermal dendritic cells) and subsequent Th2/Th22-mediated immune responses. Cytokines such as IL-4, IL-13, IL-31, and IL-22 drive inflammation, pruritus, and further barrier impairment. Created with BioRender.com.

Atopic dermatitis (AD) is a common chronic condition characterized by inflammation of the skin, which represents a significant burden on both healthcare systems and the patient’s quality of life. It has a high emotional impact as well as symptoms that are often debilitating because of the itching and unpredictable flare-ups. Due to these issues, medical treatments need to be more than just symptomatic relief; they also need to be effective in helping patients maintain long-term control over their disease [1]. AD develops as a result of multiple interacting factors, including immunologic dysregulation, environmental factors that contribute to stress, damage to the skin barrier (i.e., dry skin) and genetic predisposition, leading to a loss of normal homeostasis in the skin (Figure 1). Mutations to the gene coding for filaggrin and alterations to lipids compromise the structure of the epidermis and permit allergens and bacteria to penetrate through the skin via the epidermal barrier [2]. A failure of the skin barrier to function properly establishes dysfunction at the interface between the barrier and the immune system, which is central to the pathophysiology of AD. The breakdown of the skin barrier results in dysregulation of the type 2 immune response which primarily includes T-helper (Th) 2 and Th22 cytokines (IL-4, IL-13, IL-31, and IL-22), resulting in a cycle of ongoing inflammation. Calcineurin-dependent signaling is a key contributor to T-cell-mediated inflammation in atopic dermatitis. Activation of this pathway promotes nuclear factor of activated T cells (NFAT)-driven transcription of pro-inflammatory cytokines central to the Th2-skewed immune response. Dysregulated calcineurin activity therefore represents a mechanistically relevant node in AD pathogenesis, providing a strong biological rationale for therapeutic targeting through calcineurin inhibition [3].

In addition to prolonging inflammation, these cytokines further inhibit the ability of the skin to heal after suffering a loss of integrity to the epidermal barrier. Immunological activation caused by superantigens is increased with IMD from microbiome dysbiosis, particularly *S. aureus* overgrowth. Environmental and psychological triggers like stress, pollution and humidity cause changes to keratinocyte and immunological function as epigenetic modulators [3,4]. Thus, atopic dermatitis (AD) is considered an immunological disorder with both environmental and genetic elements communicating through molecular crosstalk to dictate its initiation, duration and response to therapies.

Although major advances in targeted systemic therapies, including biologics and Janus kinase (JAK) inhibitors, have transformed the management of moderate-to-severe AD, their emergence has also raised important questions regarding the ongoing role of established topical immunomodulators. In particular, it remains unclear how topical calcineurin inhibitors should be positioned relative to newer agents with respect to long-term disease control, site-specific treatment needs, safety, accessibility, and cost. Addressing this gap requires not only an assessment of efficacy, but a critical evaluation of where CNIs continue to provide distinct clinical value within contemporary treatment algorithms [5,6].

Calcineurin inhibitors were initially developed as systemic immunosuppressive agents, with cyclosporine and tacrolimus introduced for organ transplantation due to their potent inhibition of T-cell activation via the calcineurin–NFAT pathway. Recognition of the central role of this pathway in inflammatory skin diseases such as atopic dermatitis, together with concerns over systemic toxicity, led to the development of topical formulations. The introduction of topical tacrolimus and pimecrolimus represented a key advance by enabling localized, steroid-sparing immunomodulation with minimal systemic exposure [7]. Topical corticosteroids remain the first line of treatment for atopic dermatitis despite some limits of steroid-focused therapy such as skin thinning, tachyphylaxis and decreased adherence in sensitive areas. The development of topical calcineurin inhibitors (CNIs) as a different class of non-steroidal immunomodulators capable of providing specific T-cell modulation changed the way atopic dermatitis can be treated. Topical CNIs, such as tacrolimus and pimecrolimus, decrease inflammation while preserving the integrity of the skin through inhibition of calcineurin signaling and cytokine production [7,8]. This inhibition makes them appropriate for use on the face, eyelids and flexural surfaces. Even in the presence of biologics and JAK inhibitors, CNIs retain clinical importance by addressing localized, steroid-sensitive disease that is not optimally managed by systemic escalation alone [9]. Therefore, CNIs provide a therapeutic equilibrium by maintaining the trust developed over many years of clinical experience and adapting to an era of precision medicine and new therapies. Evaluation of the relevance of CNIs in advancing patient outcomes and achieving long-term disease control must continue by integrating them with newer treatment modalities [10,11].

The long-term safety and the absence of steroid-related adverse health effects supports CNI use in long-term AD management and in areas of delicate skin where corticosteroid therapy is less desirable. CNIs represent an outstanding therapeutic option in restoring and maintaining AD in patients who have failed conventional topical agents and experience recurrent AD flares. In contemporary treatment algorithms, CNIs occupy an intermediate position relative to conventional topical corticosteroid agents and advanced systemic therapies such as biologics and Janus kinase (JAK) inhibitors, providing a balanced therapeutic option for patients who are either seeking continued long-term disease control or have recurrent disease flares as a result of continued use of corticosteroids [12,13,14].

CNIs offer an immunomodulatory, site-specific and non-steroidal solution without the financial constraints of sophisticated biologics. Their function as a bridge therapy is especially crucial for patients who need long-term care, have steroid-sensitive skin areas, or experience frequent flare-ups. Furthermore, they are regarded as trustworthy drugs in both pediatric and adult populations due to their substantial post-marketing safety data and wide clinical experience [15].

Recent clinical guidelines reinforce the role of topical calcineurin inhibitors (TCIs) as integral to the management of AD. Proactive TCI use as part of a longer-term management plan for children with AD has been recommended by the American Academy of Pediatrics (AAP, 2025) due to their safety in sensitive areas and lack of steroids. TCIs are placed at the forefront of stepwise management of chronic AD when topical corticosteroid (TCS) use is limited due to the risk of dermal atrophy or growth status changes [16,17]. Patients in primary care also provide more evidence to support this notion; in a 2026 evidence review in the *Canadian Family Physician*, TCIs were considered effective for treating chronic AD, even with burning and pruritus being common concerns with tolerability. These collective viewpoints illustrate that TCIs should no longer be viewed as alternative therapies to first-line treatments but should be included as guideline-supported adjunctive therapy to first-line therapies within individualized treatment algorithms [18,19,20].

This review aims to critically evaluate the role of CNIs within the AD treatment continuum, bridging conventional corticosteroid therapy and emerging systemic approaches. This work attempts to demonstrate how CNIs can be employed with individualized treatment strategies by looking at their mechanisms, clinical uses, comparative benefits and integration with newer modalities. The ultimate objective is to highlight their ongoing significance in the current era of managing AD, making sure that treatment choices strike a balance between patient-centered outcomes, safety, efficacy and accessibility. This manuscript explores the role of CNIs in AD therapy, examining their historical significance, mechanistic rationale, clinical utility and future relevance amidst the rapidly shifting therapeutic landscape.

## 2. Methodology

A targeted narrative literature review was conducted to identify relevant studies evaluating the role of topical calcineurin inhibitors (CNIs) in atopic dermatitis. Electronic searches were performed using the PubMed/MEDLINE, Scopus, and Web of Science databases. The search strategy combined keywords and Medical Subject Headings (MeSH) including atopic dermatitis, eczema, topical calcineurin inhibitors, tacrolimus, pimecrolimus, biologics, JAK inhibitors, proactive therapy, and steroid-sparing treatment. Articles published primarily between 2000 and 2025 were considered, reflecting the period following the clinical introduction of topical CNIs through to the current biologic era. Priority was given to randomized controlled trials, systematic reviews, meta-analyses, clinical guidelines and large observational or registry studies. Preclinical and formulation-focused studies were included where they provided mechanistic or translational relevance. Studies were selected based on the relevance to clinical efficacy, safety, long-term use, positioning within treatment algorithms, and comparative context with emerging systemic therapies. The aim was to synthesize clinically meaningful evidence that informs on the evolving role of CNIs within contemporary atopic dermatitis management.

## 3. Pathophysiological Rationale for Calcineurin Inhibition

**Figure 2:**
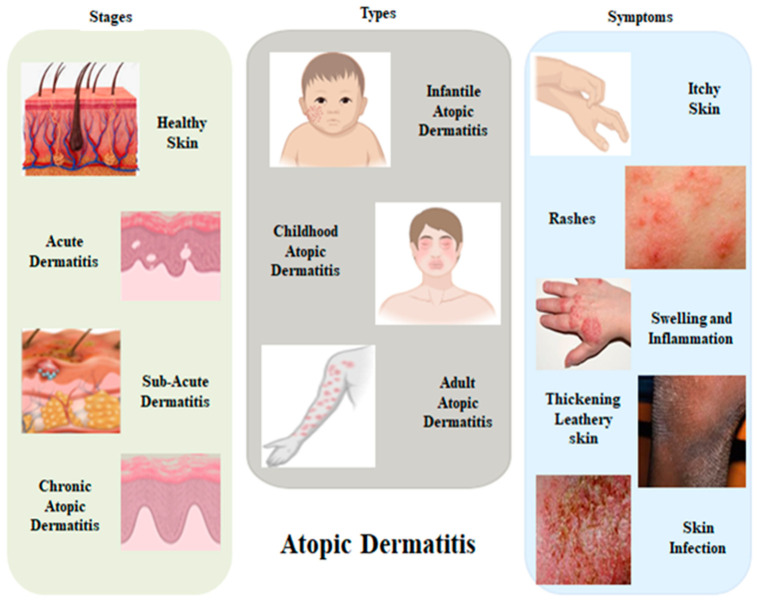
**Stages, types and symptoms of atopic dermatitis.** Created with BioRender.com.

Atopic dermatitis is caused by a complicated interaction of immunological dysregulation, dysfunction of the epidermal barrier and genetic predisposition. The abnormal activation of T cells (particularly those polarized into Th2 lymphocytes) that use cytokines (IL-4, IL-13 and IL-31) to induce and perpetuate chronic inflammation is at the center of the process of atopic dermatitis [21]. These mediators are responsible for maintaining microbial colonization, causing barrier damage, and also causing pruritus and eczematous lesions. The clinical variants, pathological stages and common symptoms associated with atopic dermatitis are illustrated in Figure 2.

### 3.1. Role of T-Cell Activation and NFAT Pathway in AD and Its Inhibition by CNIs

The calcium/calmodulin-dependent phosphatase calcineurin is essential for T-cell receptor signaling. Members of the nuclear factor of activated T cells (NFAT) family are dephosphorylated by calcineurin upon antigen stimulation, which permits them to migrate into the nucleus and start producing pro-inflammatory cytokines such as IL-2 and other Th2 cytokines [22].

Tacrolimus and pimecrolimus are examples of CNIs that work by attaching to intracellular immunophilins like FK506-binding protein 12 (FKBP12) and creating a complex that suppresses calcineurin action specifically. CNIs inhibit downstream transcription of IL-2 and other cytokines essential for T-cell proliferation and Th2-skewed inflammation by blocking NFAT dephosphorylation [23]. Under normal conditions, dephosphorylated NFAT translocates into the nucleus and promotes the transcription of multiple pro-inflammatory cytokines including IL-2, IL-4, IL-13, IL-31, and IFN-γ. By maintaining NFAT in its phosphorylated, cytoplasmic state, CNIs effectively suppress T-cell activation and downstream cytokine production, leading to attenuation of local inflammatory signaling in atopic dermatitis [16,17].

This mechanism is distinct in that it selectively modulates immune activation without inducing keratinocyte apoptosis, dermal collagen suppression, or epidermal thinning, effects commonly associated with prolonged topical corticosteroid use. As a result, CNIs preserve skin barrier integrity while exerting targeted immunosuppression, making them particularly suitable for chronic application on steroid-sensitive areas such as the face, eyelids, and intertriginous regions [22,23].

In contrast, emerging systemic therapies for atopic dermatitis intervene at different points within the inflammatory cascade. Biologic agents such as dupilumab and tralokinumab act extracellularly by neutralizing IL-4 and IL-13 signaling, thereby broadly suppressing type 2 inflammation across multiple immune compartments [24]. Janus kinase inhibitors function downstream of cytokine receptors by inhibiting JAK–STAT signaling pathways involved in cytokine signal transduction. While these approaches provide robust systemic disease control, they lack site specificity and are associated with systemic immunomodulation and monitoring requirements. CNIs, by acting upstream at the level of T-cell activation and remaining largely localized to the skin, occupy a mechanistically distinct and complementary niche rather than being rendered obsolete by newer therapies [25].

**Figure 3:**
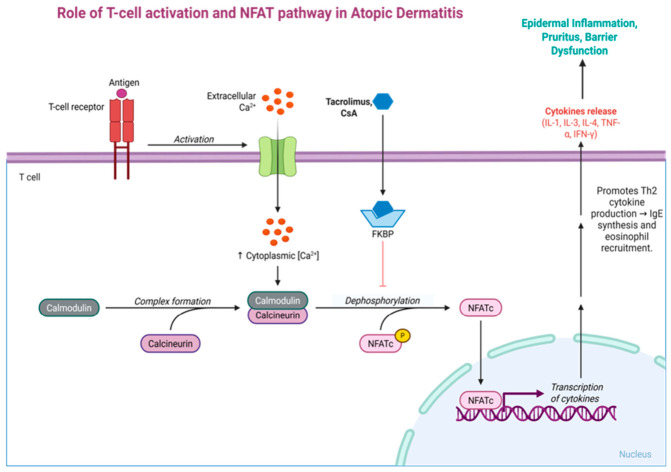
**Role of T-cell activation and the NFAT pathway in atopic dermatitis and its inhibition by CNIs.** Activation of the T-cell receptor (TCR) increases intracellular Ca^2+^ levels, leading to calmodulin-mediated activation of calcineurin. Activated calcineurin dephosphorylates NFAT, enabling its nuclear translocation and transcription of Th2 cytokines (IL-4, IL-5, IL-13, and IL-31), which drive inflammation and pruritus in AD. CNIs such as cyclosporine A and tacrolimus prevent NFAT activation by blocking the calcineurin–calmodulin complex, thereby reducing cytokine expression and immune-mediated skin inflammation. Created with BioRender.com.

Importantly, one of the main advantages of using CNIs in long-term treatment, particularly in delicately involved facial and skin folds, is that they avoid inducing cutaneous atrophy while allowing normal collagen formation to occur (as compared to corticosteroids). Thus, inhibiting the calcineurin–nuclear factor of activated T cells (NFAT) signaling pathway provides an excellent approach to inhibiting the pathological activation of T-cells occurring during AD, making CNIs an ideal choice of targeted therapy for AD [24]. Within the broader immune signaling cascade of atopic dermatitis, therapeutic interventions act at distinct but complementary levels. Calcineurin inhibitors suppress T-cell activation through inhibition of NFAT signaling, whereas biologics target upstream cytokine pathways such as IL-4 and IL-13, and JAK inhibitors block intracellular cytokine signal transduction via the JAK–STAT axis [25]. This signaling cascade maintains the inflammatory cycle that characterizes AD and increases immune activation as depicted in Figure 3.

### 3.2. Chemical Structure Aspects of CNIs

CNIs such as tacrolimus and pimecrolimus belong to the macrolactam family of natural or semisynthetic compounds, characterized by large cyclic lactone structures with embedded functional groups that confer lipophilicity and selective immunomodulatory activity. Tacrolimus is a 23-membered macrolide lactam derived from *Streptomyces tsukubaensis*, containing multiple oxygen heteroatoms, conjugated double bonds, and a high degree of hydrogen bond-donating/accepting sites, which facilitate its tight binding to the immunophilin FK-binding protein-12 (FKBP12) [25]. Structurally, the CNI complex has a shape that allows it to form a ternary complex with calcineurin, creating a very potent inhibitor of the calcineurin–NFAT signaling pathway.

Pimecrolimus is a synthetic metabolite of an ascomycin and is a macrolactam compound that is closely related in chemical structure to tacrolimus; however, the addition of a methoxyphenyl group at the C-33 position (3-O-alkylsomycin) increases the lipophilicity and tissue selectivity while decreasing the systemic absorption following topical application, thereby favoring its preferential fractional distribution within the epidermis and dermis, making it suitable for long-term administration in the facial and intertriginous areas [26].

The CNI macrolactam scaffold produces a relatively high molecular weight due to its molecular contour and resultant structures, which give rise to the conformational stability and selective protein–protein binding capabilities of CNIs that set them apart from corticosteroids and allow for localized immunosuppression and for CNIs to cause no atrophic changes [27]. From a pharmacokinetic perspective, the topical behavior of tacrolimus and pimecrolimus is shaped not only by their shared calcineurin-targeted pharmacology, but also by their physicochemical properties and the integrity of the skin barrier. Tacrolimus is relatively more lipophilic and is generally associated with stronger local skin retention, whereas pimecrolimus, because of its structural modification and cream-based formulation, is more suitable for superficial, sensitive, and intermittently inflamed sites. In intact skin, both agents show limited systemic absorption; however, in atopic dermatitis, where barrier disruption increases transepidermal flux, penetration becomes more clinically relevant. This distinction helps explain why tacrolimus is often favored for more active or refractory lesions and pimecrolimus for milder disease in delicate areas [25,26].

**Figure 4:**
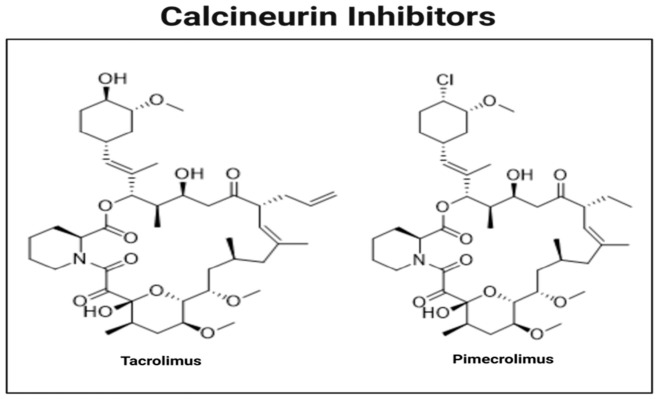
**Structures of calcineurin inhibitors, i.e., tacrolimus and pimecrolimus.** Created with BioRender.com.

Molecular engineering with nanoformulations and pro-drug approaches continues to utilize these structural properties to develop formulations that will enhance skin penetration, stability and directed delivery. Chemical structures of CNIs are depicted in Figure 4.

## 4. Clinical Evidence for CNIs

Tacrolimus 0.1% demonstrates efficacy comparable to mid-potency topical corticosteroids on the trunk and limbs, while offering the advantage of avoiding skin atrophy, making it particularly suitable for delicate areas such as the face and flexural regions [28,29]. On non-hyperkeratotic sites, CNIs demonstrate superior efficacy compared with vehicle and comparable global efficacy to low-to-mid potency corticosteroids, with fewer steroid-related local adverse effects such as atrophy and telangiectasia [30,31]. According to several meta-analyses, tacrolimus (0.03% and 0.1%) and pimecrolimus (1%) had higher short-term clearance rates and are better than the vehicle for pruritus and overall improvement as judged by patients and doctors [32,33]. A quick comparative overview of CNIs (tacrolimus and pimecrolimus) is given in Table 1.

### 4.1. Analysis of Key RCTs and Meta-Analyses for Tacrolimus and Pimecrolimus

Systematic evaluations over the past two decades have substantially clarified the clinical efficacy of topical CNIs in AD. Early meta-analyses, such as those by Ashcroft et al. (2005) and Schmitt et al. (2011), consistently demonstrated that tacrolimus outperforms pimecrolimus and placebos, with efficacy comparable to moderate-potency topical corticosteroids (TCSs) [43,44]. These results indicate that tacrolimus could act as an alternative medication when it is not appropriate to use steroids over long periods of time. Both the reviews conducted by Svensson et al. (2011) and Drucker et al. (2018) confirm the use of tacrolimus over steroids in moderate-to-severe cases of AD, but they also outlined the limitations of pimecrolimus for treating children who have mild-to-moderate forms of AD [10,45].

A significant focus of research on TCIs has been on safety. The reviews published by Ashcroft et al. (2005) and Broeders et al. (2016) acknowledged that the most prevalent adverse events associated with TCI use occur at the sites of application (i.e., a burning sensation or itchiness); however, they also acknowledged that most of these adverse events will decrease with continued use of that particular TCI [43,47]. In addition, TCIs do not cause skin atrophy like corticosteroids, which supports their preferential use on “delicate” areas of skin (i.e., the face, eyelids, and in the skin folds between body parts). There is also strong evidence from Wollenberg et al. (2021) and Jedlowsk et al. (2019) to suggest that long-term use of TCIs has been well tolerated, providing additional support for the use of TCIs in proactive or sequential treatment plans [48,49].

The literature has ongoing debate and discussion related to the possibility of TCIs causing cancer. Early indication from epidemiology work raised concern about the potential for malignancy; however, rigorous meta-analyses by Hui et al. (2021); Siegfried et al. (2019); and Margolis et al. (2015) consistently showed no effect on cancer risk including in the pediatric population [50,51,52]. A few reviews have suggested an association between TCIs and lymphoma (Kim et al., 2019); however, most of the associations were attributed to confounding by underlying disease severity rather than to a direct causal pathway [50,51,52,53,54]. Importantly, the absolute incidence of malignancy is known to be extremely low (<0.1%) indicating that if TCIs are used appropriately, there is no clinical risk associated with these agents.

In addition to safety, the available data have consistently demonstrated quality-of-life benefits associated with the use of TCIs. Eichenfield et al. (2012), Kim et al. (2019) found that the use of TCIs resulted in significant improvements in pruritus, sleep and other patient-reported outcomes. Improvement in those outcomes was even greater if TCIs were initiated proactively or in combination, such as using dupilumab and tacrolimus in preschool children. The rates of disease control achieved when using such combinations were superior to those achieved with monotherapy [54,55,56]. The evolving evidence reinforces that TCIs should be considered an essential component of long-term disease management, not just reactive therapies.

Network and comparative meta-analyses have defined the types of therapeutic relationships available to treat patients’ conditions (Drucker, 2018); however, the effectiveness of these treatments and their respective place in newer biologic or small molecule treatments must consider the effects on the body and their steroid-sparing ability [57,58]. In addition, proactive and sequential direction from Eichenfield et al. (2012) and Wollenberg et al. (2020) confirms the continued importance of TCIs within the biologic era as a means to provide the greatest clinical benefit (remission) while reducing the burden of using steroids [48,55].

The effectiveness of TCIs for treating AD has been shown in multiple systematic reviews and clinical trials, which found tacrolimus (0.1%) to be one of the most successful topical agents (in terms of atopic dermatitis) after two years and similar to both topical potent corticosteroids and topical JAK inhibitors. However, it had more transient application site reactions. Similarly, based upon the results of a 2025 network meta-analysis published in *Dermatology and Therapy*, tacrolimus was among the most effective treatments for atopic dermatitis; therefore, its use remains important even as new products are developed [59]. A meta-analysis evaluating interventions for the treatment of pruritus (Frontiers in Medicine, 2022) illustrated that TCIs lead to a statistically significant reduction in the severity of itching relative to a vehicle (i.e., a placebo), thereby improving the quality-of-life for patients with atopic dermatitis. Additionally, a prospective randomized controlled trial (RCT) of 96 children with atopic dermatitis conducted in 2024 by *Frontiers in Medicine* demonstrated that tacrolimus is superior to hydrocortisone with regards to reducing inflammatory biomarkers and clinical severity, which provides mechanistic supporting evidence for its efficacy clinically [60,61].

The evidence that has been collected from over 40 systematic reviews and meta-analyses indicates TCIs are effective and have a favorable safety profile, and supports that TCIs are essential in the treatment of AD. Tacrolimus exhibits a greater degree of efficacy in the management of moderate-to-severe disease and pimecrolimus offers a less potent but still valuable treatment for mild AD in children. The concerns regarding the risk of malignancy are generally overstated, but close surveillance of patients using TCIs is still warranted. Furthermore, TCIs have been shown to improve both the objective measurement of disease activity and the patient’s quality-of-life, thereby reinforcing their role as long-term maintenance treatments. The future of TCIs will involve the integration of TCIs with biologics and JAK inhibitors, an area of great promise, and future research should focus on optimizing combination therapies, appropriate therapy based on patient phenotype and prioritizing patient-reported outcomes [62,63,64,65]. A summary of key clinical studies, systematic reviews and meta-analyses that support these conclusions is provided in Table 2.

### 4.2. Efficacy of Tacrolimus and Pimecrolimus in Facial and Flexural AD Where Corticosteroids Pose Limitations

Tacrolimus and pimecrolimus, within a group of medications known as CNIs, represent an important treatment option for AD, particularly for sites where topical corticosteroids have demonstrated safety concerns. Their efficacy in treating lesions on the face and skin folds that are at risk of steroid-related side effects (such as skin thinning) supports their role as effective steroid-sparing therapies [95]. Compared with topical corticosteroids, CNIs can be used safely on delicate skin for long periods of time while providing effective ongoing control of disease, leading to greater improvement in patient’s overall wellbeing.

Furthermore, real-world data that complements clinical trial findings (through typical patterns of effectiveness and adherence) shows that both children and adults commonly tolerate CNIs well, based upon findings from longitudinal registries and observational studies, and the favorable safety profiles of CNIs facilitate their longer-term use [96,97,98]. High levels of adherence due to decreased concern about skin thinning and ongoing consistent disease control are often reported by chronic and relapsing patients treated with CNIs. Taken together, these insights emphasize that beyond controlled clinical settings, CNIs remain highly relevant in bridging efficacy, safety, and patient-centered outcomes, reinforcing their continued role in modern therapeutic strategies for AD [99].

## 5. Safety Profile and Controversies of CNIs

### 5.1. Common Adverse Effects of CNIs

Transient application-site burning/stinging, pruritus, erythema, and warmth are the most common side effects; these usually peak in the first few days of treatment and then go away as the barrier gets better. Rather than representing drug toxicity, this neuro-sensory stinging is believed to result from the exposure of cutaneous nerve endings in inflamed, barrier-disrupted skin. The reaction often diminishes when topical CNIs are initiated after a short corticosteroid “lead-in” on highly inflamed plaques [99,100]. When applied correctly externally, periocular usage rarely results in true ocular problems; however, it may produce brief discomfort. Tacrolimus is known to cause alcohol-flush reactions, which are brief flushes of the face following alcohol use; counseling can prevent needless stopping.

TCIs are recommended for thin-skin locations (facial, folds, genital, and eyelid) and for long-term “proactive” maintenance on previously active sites since, in contrast to topical steroids, they do not result in dermal atrophy, striae, telangiectasias, or tachyphylaxis. These arguments are supported by the American Academy of Dermatology (AAD) guideline overviews and FDA TCI labeling [101].

Controlled trials indicate that TCIs do not increase the incidence of routine bacterial skin infections, as they act as immunomodulators rather than broad immunosuppressants and exhibit minimal systemic absorption through intact skin [102]. Eczema herpeticum, or herpes outbreaks, is a comorbidity of AD; caution is necessary rather than complete avoidance. Although the topical dose has not been shown to cause photocarcinogenicity in humans, routine photoprotection is nevertheless recommended, mostly as a precaution and since phototherapy is frequently administered to AD patients [103]. In situations where there are significant barrier deficiencies (such as Netherton syndrome), where absorption may be increased, exercise caution. These subtleties are covered in full in the guidelines and review summaries [104].

Although application-site burning and stinging are the most common reasons for early discontinuation, these reactions are usually transient and often improve with continued use. The risk and intensity of irritation appear to be greater when treatment is started on highly inflamed, barrier-disrupted skin, so a short topical corticosteroid lead-in in very active lesions can be useful before transitioning to a calcineurin inhibitor for maintenance. In practice, concomitant emollient use should be encouraged to support barrier recovery, reduce irritant symptoms, and improve tolerability. Patient counseling is also essential, because early burning is expected, self-limited, and should not be interpreted as drug failure. For tacrolimus, clinicians should also counsel patients about the uncommon alcohol-flush reaction, which can otherwise lead to unnecessary discontinuation [99,100,101].

### 5.2. Long-Term Safety and the Black-Box Malignancy Warning for CNIs

High-dose animal studies, rare post-marketing case reports, and class concerns extrapolated from systemic CNIs in transplant medicine where the drug exposure is orders of magnitude higher than in topical use for AD formed the basis of the U.S. FDA’s black-box warning on TCIs in 2006. This decision, largely precautionary, quickly became one of the most debated issues in dermatology [99,105].

Although the FDA black-box warning was precautionary, the long-term evidence base provides little support for a causal malignancy signal with topical calcineurin inhibitors. In the APPLES prospective pediatric cohort, 7954 children were followed for 44,629 person-years, with six confirmed cancers and no lymphomas, and the observed incidence was consistent with background expectations. The PEER cohort similarly found no significant association between pimecrolimus exposure and malignancy. Large observational data from the JOELLE study also found little evidence linking topical tacrolimus or pimecrolimus to skin cancer or lymphoma, although residual confounding by indication and disease severity cannot be excluded [92].

In the years since, extensive real-world evidence has consistently failed to confirm these early theoretical concerns. Multiple large-scale observational cohorts, disease registries, and meta-analyses demonstrate that TCIs do not increase the overall risk of malignancy. The presence of a marginal lymphoma indicated in some studies is likely due to the severity of AD, which is independently linked to a greater incidence of lymphoma, rather than the effect of using TCIs [106].

### 5.3. Evidence from Registries and Meta-Analyses of CNIs

A large amount of registry data supports this safety profile. There were more than 6800 pediatric patients followed for ten years in the Pediatric Eczema Elective Registry (PEER) who demonstrated no increase in malignancy rates with long-term treatment with pimecrolimus compared to the background population. The JOELLE international cohort (60,000 patients) of patients in four nations across Europe also demonstrated no increased risk of overall malignancies associated with tacrolimus or pimecrolimus [92]. This finding supports the conclusion that the only marginal evidence of lymphoma observed is due to the severity of the underlying condition from which patients with AD suffer, not due to the use of the medications [99,101].

Meta-analyses subsequently confirm these previous findings. A meta-analysis published in *JAMA Dermatology* in 2021 conducted an analysis of 3.4 million patient-years of use of TCI and demonstrated no relationship between the use of TCI and an increase in the risk of melanoma, keratinocyte carcinoma, or risk of overall malignancy [68]. Most recently, a meta-analysis published in *The Lancet* in 2023 demonstrated similar findings among the pediatric population [67].

The most balanced interpretation of the literature is that the overall and skin-cancer risk does not appear increased, while pooled analyses have reported a small lymphoma signal that is likely to have limited absolute clinical impact and may reflect confounding by atopic dermatitis severity rather than a direct drug effect. Accordingly, TCIs should be discussed as agents with a reassuring long-term safety record, but with continued pharmacovigilance and transparent acknowledgement of the remaining uncertainty in high-risk populations [106].

The cumulative information being gathered on TCIs from registries, cohorts and meta-analyses have all supported their use as safe and effective steroid-sparing options for both adults and children alike. Although continuing pharmacovigilance is important, many current global guidelines, including those from the European Task Force on AD and AAD Guidelines (2023), now claim TCIs as the mainstay treatments for moderate-to-severe disease, especially in areas of sensitive skin [99].

## 6. Comparative Effectiveness: CNIs vs. Newer Therapies

### 6.1. CNIs vs. Biologics

The TCIs tacrolimus and pimecrolimus are used for localized, non-steroidal immunosuppression by inhibiting T cell activation through the NFAT pathway. Major benefits of TCIs include localized delivery, avoidance of topical corticosteroid-induced skin atrophy and acceptable use on sensitive skin areas like the face and intertriginous regions [107]. Both of these agents are used for the treatment of mild-to-moderate AD and require continued long-term use to maintain remission from AD.

**Figure 5:**
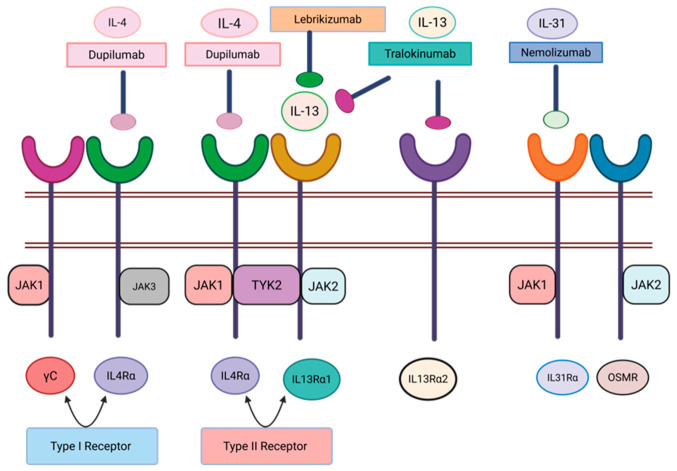
**Mechanism of action of biologics targeting IL-4 and IL-13 signaling pathways in atopic dermatitis.** This schematic illustrates the interleukin (IL)-4 and IL-13 signaling pathways involved in type 2 inflammation and highlight the molecular targets of approved biologics used in AD management. IL-4 and IL-13 interact with their respective Type I and Type II receptor complexes, which include IL-4Rα, IL-13Rα1, and γC, activating downstream JAK–STAT signaling via JAK1, JAK2, JAK3, and TYK2. Created with BioRender.com.

Biologics like dupilumab and tralokinumab have an impact on the treatment of AD by targeting the IL-4Rα/IL-13 signaling pathway, one of the main pathways involved in type 2 inflammatory disease (Figure 5). Monoclonal antibody products provide systemic control of moderate-to-severe AD that is inadequately treated with topical therapy, decreasing symptoms of eczema, decreasing pruritus and improving the quality of life for the patient [108]. Although biologics are more costly and delivered by injection, multiple clinical trials (LIBERTY AD and ECZTRA) have demonstrated the greater efficacy and durability of the clinical response with biologics compared to standard topical therapy [109].

To strengthen the evidence base, this review integrates findings from recent high-level evidence syntheses. A Cochrane Network Meta-Analysis (2024, Clinical & Experimental Allergy) compared various topical treatments (TCIs and steroids) and newer topical medications (JAK inhibitors and PDE4 inhibitors), showing that TCIs are very effective for eczema. This analysis is further supported by a systematic review of the literature and network meta-analysis (2025, Dermatology & Therapy), which included newer topical medications and also found TCIs to be one of the most effective topical treatments (Tacrolimus) [81,88]. Additionally, a scoping review was conducted (2022, Dermatology & Therapy) which explored the current understanding of the use of proactive therapy to prevent flares, specifically examining TCIs [90,108]. These studies together provide an overview of the current evidence available for both classical RCTs as well as newer types of comparisons.

From a clinical point of view, CNIs are still an important adjunct therapy to steroids for use in steroid-responsive areas and children, whereas biologics provide the ability to change the overall systemic disease of widespread or resistant eczema. This difference highlights a two-tier treatment method in which the CNIs act as a treatment locally modulating the immune system, while the biologics treat the systemic nature of the disease [110,111].

#### 6.1.1. CNIs as Precision Topicals: Targeting Sensitive Skin Regions

CNIs are useful for treating AD at specific sites where long-term corticosteroid use is limited by atrophy of the skin. Topically administered CNIs achieve targeted immunoregulation of the skin in sensitive areas (face, eyelids, and intertriginous areas), making them ideal for children and maintaining long-term therapy. In contrast, biologic agents like dupilumab and tralokinumab work systemically and are most appropriate to treat moderate-to-severe AD throughout the body [112]. Therefore, CNIs are essential for the treatment and control of localized flares in sensitive and delicate skin due to safety and irritable properties.

#### 6.1.2. Cost, Accessibility and Pediatric Use of CNIs and Biologics

Biologics and JAK inhibitors have transformed the paradigm for treating moderate-to-severe AD, but their high cost, parenteral route and limited widespread use in the healthcare system, particularly in low- and middle-income countries, have hindered their widespread use. However, TCIs like tacrolimus and pimecrolimus remain accessible, inexpensive and simple to use, providing a useful therapy option in a range of healthcare systems. Pediatric registries provide strong evidence for their long-term safety profile, highlighting their significance in youngsters where reducing corticosteroid-induced atrophy is a top priority [113]. Additionally, CNIs are especially beneficial for anatomically sensitive areas like the face, eyelids and intertriginous tissues where long-term corticosteroid administration is discouraged. Therefore, CNIs continue to have therapeutic significance as steroid-sparing, site-specific and economical medicines and more sophisticated treatments even in the age of precision biologics [114].

### 6.2. CNIs vs. JAK Inhibitors

JAK inhibitors are a new type of drug that has improved AD treatment by blocking signaling from various amounts of cytokine receptors, including IL-4, IL-13 and IL-31. This blockage results in rapid improvement in pruritus and systemic disease control, frequently within days of treatment initiation [115]. Clinical trials involving baricitinib, upadacitinib and abrocitinib consistently show good efficacy in moderate-to-severe AD with marked improvements in EASI and IGA scores.

However, there are safety issues with systemic JAK inhibition, like thromboembolic events, serious infections, herpes zoster reactivation and laboratory abnormalities such as lipid elevations and cytopenias. As a result, regulatory bodies like the FDA and EMA have stressed the need for cautious use, especially in patients who have cardiovascular risk factors or other vulnerabilities [116].

Topical CNIs do not result in systemic immunosuppression and instead operate locally within the skin. They are useful for localized AD because of their good long-term safety profile and absence of need for laboratory testing, especially in patients that are not candidates for systemic therapy, as well as in pediatric populations and settings with limited resources [117,118]. Consequently, CNIs continue to be an efficient and safer option for treating limited types of AD, while JAK inhibitors increase the therapeutic options for severe illness.

### 6.3. Integrative Therapeutic Ladder

These days, CNIs are an essential component of an integrative therapy ladder that links traditional and innovative treatments for AD. Despite the fact that biologics and JAK inhibitors have revolutionized the treatment of systemic illness, their use is often limited to moderate-to-severe AD but impeded by cost, safety and monitoring. CNIs remain crucial as first-line topical steroid-sparing drugs, particularly in sensitive areas including the face, eyelids and intertriginous zones, where corticosteroid use is forbidden [119].

CNIs serve as a therapeutic link between systemic immunomodulators and conventional corticosteroids. Their proven safety profile, efficacy in localized disease and suitability for both pediatric and adult patients make them an accessible therapeutic link. For many patients, CNIs provide long-lasting management of recurrent flares without the risk of skin atrophy, delaying or even eliminating the need for systemic escalation [110,120].

In the current treatment landscape for AD, CNIs can best serve as a maintenance therapy for patients experiencing an exacerbation of atopic dermatitis after a course of corticosteroids, until the patient may need a therapy such as a biologic or JAK inhibitor. This integrated approach is consistent with the principles of precision medicine, which emphasize matching the severity of the disease with the intensity of the therapy and allocating resources wisely, particularly in healthcare systems that face budgetary constraints [121]. CNIs are established as a first-line therapy in steroid-sparing treatment algorithms, demonstrating their continued therapeutic value in the evolving area of AD treatments. Table 3 shows a comparative landscape of topical CNIs, biologics, JAK inhibitors and innovative CNI delivery systems in AD, emphasizing the site of action, onset, efficacy, safety, pediatric applicability, cost, combination strategies and future prospects.

### 6.4. Combination Therapy and Maintenance Use of CNIs with Biologics

Combination therapy is increasingly relevant in contemporary atopic dermatitis care, particularly when biologics are used for systemic disease control while topical calcineurin inhibitors are retained for residual, site-specific inflammation in sensitive areas. This strategy is consistent with the way TCIs are positioned in guideline-based topical management, where tacrolimus or pimecrolimus may be used when corticosteroids are not suitable, fail to work, or are undesirable for long-term use. In practice, this creates a rational maintenance model: biologics reduce the global inflammatory burden, while TCIs provide localized steroid-sparing control on the face, eyelids, flexures, and other recurrent flare sites [73,74].

The evidence base for biologic–TCI combination therapy is still emerging and should be presented as such. A phase III dupilumab trial with concomitant topical corticosteroids showed significant improvement in signs, symptoms, and quality of life, supporting the broader principle that systemic biologics are often used with topical anti-inflammatory support rather than as complete replacements for topical therapy [82]. More recently, a 2025 pediatric study reported that dupilumab combined with topical calcineurin inhibitors improved the symptoms and quality of life in preschool children with moderate-to-severe AD [84]. However, direct comparative trials specifically testing biologic plus TCI maintenance against biologic monotherapy remain limited, so the role of TCIs should be framed as a complementary, phenotype- and site-driven strategy rather than a universal standard [117,118].

### 6.5. Emerging Topical and Systemic Modulators in AD

Recent phase II/III trials have expanded the therapeutic landscape of atopic dermatitis beyond corticosteroids, CNIs, biologics, and conventional JAK inhibitors. Among topical agents, cream has shown meaningful anti-inflammatory and antipruritic efficacy in phase 3 studies, with sustained disease control and favorable tolerability in longer-term follow-up, including supportive pediatric data [130]. Delgocitinib ointment has also demonstrated a clinical benefit in pediatric and infant atopic dermatitis in phase 3 and long-term studies, reinforcing the growing role of topical cytokine pathway modulators as steroid-sparing alternatives [131].

On the systemic side, lebrikizumab phase 3 trials demonstrated rapid and clinically meaningful improvement in moderate-to-severe atopic dermatitis, including adolescent populations, while nemolizumab phase 3 studies showed significant reductions in inflammation and itch as a monotherapy and when combined with topical therapy [132,133]. Long-term extension data further support the durability of response and tolerability of these newer systemic modulators.

Although these newer agents broaden treatment options, much of the evidence remains placebo-controlled or extension-based, and direct head-to-head comparisons with established topical therapies remain limited. Therefore, CNIs should be interpreted not as outdated therapies, but as complementary, site-specific treatments that remain important for sensitive areas, pediatric disease, and long-term steroid-sparing management [134].

### 6.6. Critical Synthesis: Why CNIs Remain Relevant in the Biologic Era

The persistence of topical calcineurin inhibitors in modern atopic dermatitis care reflects a therapeutic gap rather than simple historical inertia. Biologics, such as dupilumab and tralokinumab, and oral JAK inhibitors provide broader systemic disease control and faster improvement in moderate-to-severe disease, but they do not replace the need for localized, non-atrophogenic, steroid-sparing treatment in delicate skin sites. CNIs therefore occupy an intermediate but indispensable position in treatment algorithms: they are less systemic than biologics and JAK inhibitors, but more suitable than corticosteroids for chronic use on the face, eyelids, and folds. Their value is further strengthened by their lower cost, ease of use, and extensive real-world safety experience. However, the evidence base still has important limitations [113,115]. Direct head-to-head trials with biologics and JAK inhibitors are scarce, most studies have a short follow-up, proactive maintenance schedules are not standardized, and patient-reported outcomes and cost-effectiveness data remain underexplored. Future research should therefore focus on comparative effectiveness in real-world practice, optimal sequencing with newer agents, and phenotype-guided treatment selection [131,132,133].

## 7. Novel Formulations and Delivery Approaches

### 7.1. Nanocarrier-Based CNI Delivery for Enhanced Penetration and Reduced Irritation

**Figure 6:**
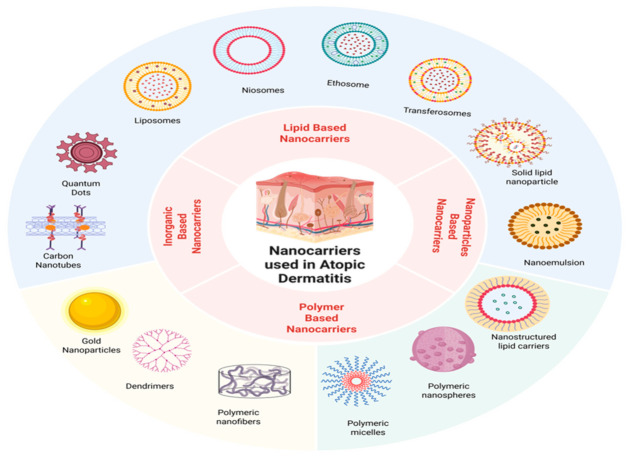
Different nanocarriers used in the treatment of atopic dermatitis. Created with BioRender.com.

The goal of next-generation topical systems is to minimize burst exposure at nerve-rich regions that cause burning or stinging while localizing CNIs within viable epidermis. Liposomes or transferosomes, ethosomes, solid lipid nanoparticles, and nanoemulsions are examples of lipid-centric carriers that facilitate delayed, prolonged release, enhance partitioning through the stratum corneum, and form epidermal depots [135]. Biomimetic shells (ceramide-rich, phosphatidylcholine or hyaluronic-acid coatings) and optimized particles (100–300 nm; near-neutral zeta potential) can (i) improve follicular and intercellular delivery, (ii) lessen irritancy by lowering Cmax at the nerve endings, and (iii) restrict systemic absorption. Pro-drug methods (esterified tacrolimus) and pH- or enzyme-responsive matrices further adjust cutaneous bioavailability with on-site activation. When combined, these designs offer improved tolerability and increased per-dose efficacy at sensitive locations (face, eyelids, and flexures) which is especially advantageous for pediatric patients and maintenance regimens [136]. Different nanocarriers used in the formulations of CNIs are depicted in Figure 6.

### 7.2. Combination Regimens: (CNI + Barrier Repair Agents)

Combining tacrolimus or pimecrolimus with advanced barrier restoration strategies significantly enhances therapeutic outcomes because barrier dysfunction increases Th2-skewing and CNI demand. The following are evidence-based add-ons: filaggrin-supportive humectants (urea and glycerol), antipruritic neuromodulators (topical menthol or polidocanol when appropriate), and multi-lamellar emollients (ceramides, cholesterol, and free fatty acids in physiological ratios) [136]. It is possible to (a) speed up lesion clearing, (b) prolong remission under proactive, twice-weekly CNI treatment, and (c) reduce cumulative steroid exposure by directly incorporating CNIs into ceramide-dominant, low-surfactant bases or by layering them with prescription-strength barrier creams [137,138].

Different nanocarrier and advanced delivery platforms for topical CNIs (tacrolimus and pimecrolimus) that are investigated in AD, summarizing the carrier types, formulation approaches, key pharmacotechnical/clinical outcomes, and supporting literature evidence is given in Table 4.

### 7.3. Device and Technique-Assisted Delivery

The therapeutic potential of CNIs is often limited by the formidable barrier properties of the stratum corneum, particularly in lichenified or treatment-refractory AD plaques. To overcome this challenge, several device- and technique-assisted approaches have been explored to transiently modulate skin permeability and enhance intradermal drug penetration [153]. Among these, dissolvable microneedle arrays represent a minimum invasive platform that bypasses the stratum corneum by creating self-resorbing microchannels that facilitate direct delivery of tacrolimus nanocrystals or polymeric carriers into the viable epidermis and dermis with the least systemic exposure. Similar to this, fractional low-energy laser systems, i.e., laser-assisted drug delivery, create regulated microthermal zones that help topical CNIs diffuse into deeper compartments to increase the efficacy in lichenified lesions. Iontophoresis and electroporation-based techniques provide a controllable and noninvasive approach to increase flux by using electrical gradients that push charged or encapsulated CNIs over the epidermis. Hydration-induced barrier disruption that passively increases penetration is achieved by therapies such as short-contact occlusion utilizing hydrocolloid or lipid-rich films [154,155].

Although these approaches demonstrate practical results, factors such as device cost, procedural competence, patient tolerance and possible dangers like infection or discomfort limit their wider clinical applicability. As a result, their application is most warranted in situations where traditional topical formulations are ineffective. Combining improved formulations with device-assisted distribution may provide a translational approach to optimize the local efficacy of nanocarrier-integrated CNIs while maintaining safety [156].

### 7.4. Formulation Quality Attributes and Safety Guardrails

Low-surfactant, alcohol-free carriers, non-sensitizing excipients, rheology that enables uniform thin-film application, low-irritancy preservation systems and lack of skin atrophy are crucial features of advanced therapies of CNIs [97]. Stability programs should confirm the content homogeneity and release kinetics at real temperatures. Clinical evaluation should prioritize patient-reported outcomes (burning or stinging within 30 min), early itching alleviation and adherence.

Smart carrier technologies in conjunction with barrier-directed therapy allow CNIs to permeate deeper where therapeutic activity is needed while reducing off-target exposure, even while biologics and JAK inhibitors expand systemic possibilities [99,109].

## 8. Practical Considerations of CNIs for Clinicians

### 8.1. Positioning CNIs in Treatment Algorithms

CNIs as first-line steroid-sparing agents for sensitive areas such as face, eyelids, intertriginous folds, and genitalia, and as second-line options for patients who are unable to tolerate or fail topical corticosteroids, are recommended by current consensus guidelines. When used two to three times a week on relapse-prone areas, they are also very well suited for long-term proactive maintenance therapy by lowering the frequency of flare-ups and cumulative exposure to corticosteroids. By offering tailored control of localized lesions, CNIs can supplement systemic therapy such as biologics and JAK inhibitors in moderate-to-severe AD [113,114].

### 8.2. Pediatric vs. Adult Use of CNIs

In pediatric AD, when long-term steroid usage includes concerns of skin atrophy, growth suppression and systemic absorption, CNIs are the first line of treatment. In many places, tacrolimus 0.03% and pimecrolimus 1% are authorized starting from age two, and real-world registries attest to their long-term clinical safety. In adults, pimecrolimus provides a milder option for early or intermittent disease, whereas tacrolimus 0.1% is preferred for more severe or refractory lesions [31,157]. They are particularly useful for chronic or relapsing diseases, since they can provide long-lasting management without causing skin thinning in people of various ages.

### 8.3. Counseling Patients for CNIs: Safety Perceptions vs. Evidence

The “black-box” malignancy warning has caused long-lasting anxiety among patients and caregivers, despite strong safety data. Clinicians should put this caution in perspective: meta-analyses and large registries indicate no elevated risk of skin cancer or lymphoma when compared to the general AD population. Patients should be advised that initial burning or itching is normal and is lessened by using emollients at the same time. The following should be emphasized in counseling: CNIs are non-steroidal, maintain the integrity of the skin barrier, and prevent atrophy; long-term use, even in children, has demonstrated a comforting safety profile; and the benefits are maximized by consistent use of emollients and adherence to proactive regimens [158].

## 9. Future Perspectives

As AD treatment enters the era of precision medicine, the function of CNIs is being redefined rather than diminished. Even if biologics and JAK inhibitors currently dominate the discussion, CNIs continue to be a uniquely positioned therapy for localized, steroid-sensitive areas such as the face, eyelids and intertriginous regions. Both monotherapy and combinatorial treatments such as the adjuvant use of systemic biologics to manage breakout flares or lessen steroid dependence in sensitive skin areas may be beneficial in the future.

The emerging international consensus underscores TCIs as central to modern AD management. Guidelines from both the American Academy of Pediatrics (AAP, 2025) and Korean AD Association (2025) converge in recommending proactive, long-term TCI use, reflecting growing confidence across regions and age groups [16,37]. The consistency of results across Cochrane (2024) and contemporary systematic reviews (2025) further confirms their comparative efficacy, though heterogeneity in study design and short follow-up durations remain limitations [88]. Biomarker-driven evidence from pediatric RCTs (2024) suggests a translational bridge between mechanistic anti-inflammatory effects and clinical outcomes, a critical step toward personalized dermatology. Large-scale cohort analyses such as the JOELLE study (Arana et al., 2021) complement RCTs by confirming safety in real-world populations, offering a more balanced risk–benefit narrative regarding malignancy [93]. Despite this robust evidence, research gaps remain in determining optimal proactive regimens, long-term comparative effectiveness with biologics or JAK inhibitors and quality-of-life endpoints. Addressing these questions will be crucial to further consolidating the role of TCIs within the evolving therapeutic landscapes [159,160,161,162].

New developments in hybrid regimens with barrier repair agents and nanocarrier formulations may further enhance adherence and tolerability, lessen local irritation and increase long-term use. Additionally, in premises with limited resources where access to biologics is still restricted, CNIs have a strategic advantage due to their affordability and widespread global availability. Thus, health-economic studies could reframe CNIs as an egalitarian, long-lasting treatment in dermatology worldwide.

**Figure 7:**
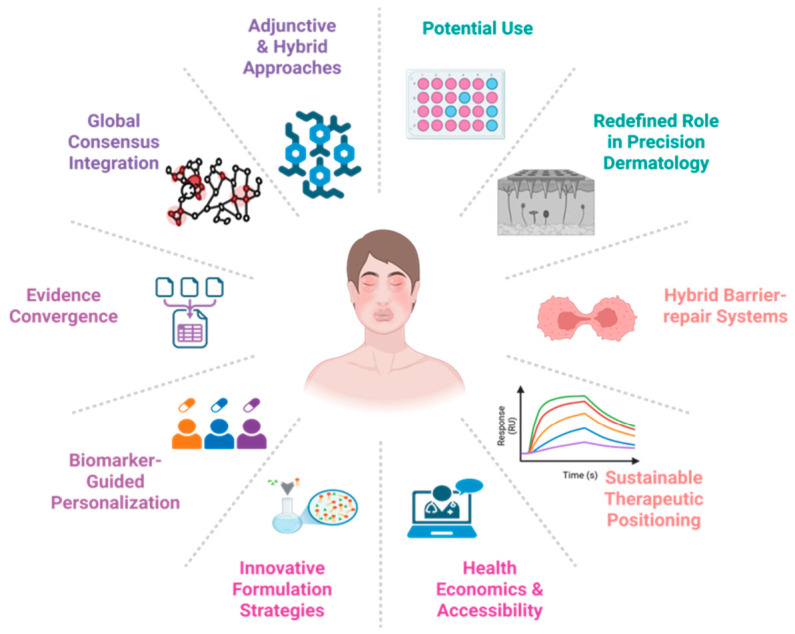
**Future perspectives of calcineurin inhibitors in atopic dermatitis.** Created with BioRender.com.

Ultimately, the future of CNIs lies in reframing them not as transitional agents of the pre-biologic era but as integral components of a multimodal, precision-based AD treatment algorithm, balancing innovation with accessibility (Figure 7).

## 10. Conclusions

CNIs continue to play a major role in the treatment of AD, particularly in delicate areas like the cheeks, eyes and skin folds. Despite the expanding therapy arsenal which includes biologics and JAK inhibitors, CNIs continue to demonstrate clinical relevance due to their well-established safety profile, local effectiveness and universal accessibility. Importantly, the sustained use of CNIs reflects not therapeutic inertia but a clearly defined clinical niche that newer systemic agents do not fully address. While biologics and JAK inhibitors provide broad disease control in moderate-to-severe AD, they are not optimized for long-term, localized management of steroid-sensitive areas, where CNIs offer a favorable balance of efficacy, safety, and practicality. Even if more recent systemic medications have revolutionized disease treatment in moderate-to-severe AD, CNIs still have unique relevance as first-line steroid-sparing topicals and as additional drugs in combo regimens. Their affordability, global accessibility and proven efficacy in both pediatric and adult populations further boost their importance in bridging cutting-edge discoveries with realistic practical therapy. Nevertheless, important evidence gaps remain, including limited head-to-head comparisons with biologics and JAK inhibitors, heterogeneity in proactive maintenance regimens, and insufficient long-term data on patient-reported outcomes and cost-effectiveness in real-world settings. In the end, CNIs serve as an example of how conventional treatments, when integrated into contemporary treatment algorithms, continue to be essential for attaining sustainable, patient-centered, and balanced AD care.

## Figures and Tables

**Table 1:** Clinical and pharmacological overview of calcineurin inhibitors, i.e., tacrolimus and pimecrolimus in atopic dermatitis.

Parameters	Tacrolimus	Pimecrolimus	Clinical Relevance	References
Molecular weight & lipophilicity	804.02 g/mol; highly lipophilic	810.48 g/mol; more lipophilic than tacrolimus	Influences penetration and site selectivity	[31,32]
Approved concentrations	0.03%, 0.1% ointment	1% cream	dosing in pediatrics and adults	[31,33]
Indications (FDA/EMA)	Moderate–severe AD, ≥2 years	Mild–moderate AD, ≥2 years	Pediatric and adult use	[33,34,35]
Common adverse effects	Burning, pruritus (transient)	Burning, pruritus (milder)	Key in patient counseling	[30,36]
RCT evidence	Significant EASI improvement vs. placebo	Early onset itching reduction, relapse prevention	Demonstrates efficacy	[37,38]
Meta-analyses	Comparable efficacy to corticosteroids, less atrophy	Effective in pediatric facial AD	Evidence-based confirmation	[39,40,41]
Long-term safety (registries)	No increase in malignancy (JOELLE registry)	No cumulative infection risk	Challenges FDA black-box warning	[42,43]
Practical use	Sensitive sites (face, eyelids); steroid-sparing	First-line in pediatric mild AD	Preferred where steroids contraindicated	[44,45,46]

**Table 2:** Summary of systematic reviews and meta-analyses evaluating the efficacy, safety, and long-term outcomes of topical calcineurin inhibitors (tacrolimus and pimecrolimus) in atopic dermatitis, with emphasis on comparative effectiveness versus corticosteroids, relapse prevention, and malignancy risk.

Sr. No.	Study	Drug	Key Findings	Clinical Implications	Ref.
1	El-Batawy et al., 2009	Tacrolimus, pimecrolimus	TCIs more effective than placebo; tacrolimus–moderately potent TCS; pimecrolimus useful as early steroid-sparing agent.	Best for chronic lesions of face/flexures; suitable for maintenance	[63]
2	Iskedjian et al., 2004	Tacrolimus vs. pimecrolimus	Both effective; tacrolimus numerically superior, used more in severe cases.	Head-to-head RCTs warranted	[64]
3	Broeders et al., 2016	TCIs vs. TCS	Similar efficacy; TCS preferred due to cost and fewer AEs.	TCS remain therapy of choice (Level-1a)	[47]
4	Siegfried et al., 2016	Tacrolimus, pimecrolimus	Robust data support long-term TCI use; limited long-term TCS safety data.	TCIs favored for maintenance; TCS for acute flares	[51]
5	Schmitt et al., 2011	Tacrolimus vs. fluticasone	Proactive use prevents flares; indirect evidence favors fluticasone.	More long-term safety trials required	[44]
6	Chen et al., 2010	Tacrolimus (0.03% vs. 0.1%), pimecrolimus	Tacrolimus superior to pimecrolimus in children; no major difference between concentrations.	Safe and effective; AEs mainly burning, pruritus	[66]
7	Devasenapathy et al., 2023	TCIs	No cancer risk in infants, children, adults.	Supports long-term safety	[67]
8	Lam et al., 2021	TCIs	Slight association with lymphoma, not other cancers.	Absolute risk remains very low	[68]
9	Wu et al., 2021	TCIs	Association with lymphoma (esp. NHL), though absolute risk small (0.02–0.09%).	Monitor high-risk patients; prospective studies needed	[69]
10	Chia & Tey, 2015	Tacrolimus, pimecrolimus	Tacrolimus comparable to TCS; malignancy risk unclear.	Tacrolimus—effective alternative; pimecrolimus weaker	[70]
11	Łabędź & Pawliczak, 2019	TCIs vs. TCS	TCIs more effective but with higher AE rates.	Better efficacy but tolerability trade-off	[71]
12	Chu DK et al., 2023	Tacrolimus, pimecrolimus, TCS	Tacrolimus, pimecrolimus and moderate TCS among most effective; antibiotics least effective.	Guides treatment hierarchy	[72]
13	Yin et al., 2011	Tacrolimus vs. pimecrolimus	Tacrolimus was more effective than pimecrolimus in achieving treatment success.	Supports tacrolimus as the preferred TCI for moderate-to-severe AD	[73]
14	Xu et al., 2025	TCIs	Dupilumab and lebrikizumab showed superior EASI-75 responses with acceptable safety.	Supports evidence-based biologic selection for moderate-to-severe AD	[74]
15	Sher et al., 2012	TCIs	Reduced pruritus by ~36% vs. vehicle.	Valid antipruritic effect	[75]
16	Dhar et al., 2023	TCS + TCI	Sequential therapy effective and well tolerated.	Important strategy in clinical practice	[76]
17	Ashcroft et al., 2005	Tacrolimus, pimecrolimus	Both > placebo; tacrolimus–potent TCS; unclear pimecrolimus role.	Role limited when mild TCSs suffice	[43]
18	Chu AW et al., 2023	TCIs	No cancer risk vs. TCS.	Actionable for guidelines	[77]
19	Chittock et al., 2024	Tacrolimus, pimecrolimus, corticosteroids	Tacrolimus showed higher efficacy, while pimecrolimus had favorable safety but lower efficacy.	Supports tacrolimus for moderate-to-severe AD and pimecrolimus for milder disease or sensitive skin areas	[78]
20	Legendre et al., 2015	TCIs	Slight lymphoma risk linked to AD severity, not TCI use.	AD severity confounder	[79]
21	Huang & Xu, 2015	Tacrolimus, pimecrolimus	Both > vehicle; similar efficacy/safety.	No major differences between TCIs	[80]
22	Lax et al., 2024	JAK inhibitors, TCS, tacrolimus	JAK inhibitors, potent TCS, tacrolimus ranked most effective.	Tacrolimus remains top-tier therapy	[81]
23	Drucker et al., 2024	Systemic immunomodulators	Upadacitinib and abrocitinib were among the most effective therapies for EASI-75, EASI-90, and IGA responses.	Supports selection of high-efficacy systemic treatments for refractory moderate-to-severe AD	[82]
24	Huang et al., 2023	TCIs	No overall cancer risk; possible ↑ leukemia in Asian AD patients.	Population-specific monitoring needed	[83]
25	Liming & Ali, 2025	Dupilumab + TCI	Combination improved symptoms in preschoolers.	Supports combination therapy	[84]
26	Draelos, 2008	TCIs	Effective without skin atrophy; mild local AEs.	Safer for sensitive skin sites	[85]
27	Zhao et al., 2023	TCIs vs. TCS	TCIs safe in children; TCSs cause atrophy.	Reinforces steroid-sparing role	[86]
28	Luger et al., 2023	Tacrolimus, pimecrolimus, mild TCS	Pimecrolimus ≈ tacrolimus 0.03% and mild TCS; no atrophy.	First-line for mild–moderate AD, esp. pediatrics	[87]
29	Martins et al., 2015	Tacrolimus, pimecrolimus, TCS, JAKi	High-certainty evidence: Tacrolimus > pimecrolimus; comparable to moderate TCS.	Positions tacrolimus as standard therapy; pimecrolimus for mild cases	[88]
30	Drucker et al., 2022	Dupilumab, baricitinib, upadacitinib, abrocitinib, cyclosporine, methotrexate, azathioprine	JAK inhibitors and dupilumab showed the highest efficacy, with differing safety profiles.	Supports selection of systemic therapies based on efficacy and safety needs in moderate-to-severe AD	[89]
31	Silverberg et al., 2022	Upadacitinib, abrocitinib, baricitinib, dupilumab, tralokinumab	Higher-dose JAK inhibitors, especially upadacitinib and abrocitinib, showed the greatest improvements in EASI and pruritus.	Supports individualized selection of targeted systemic therapies based on efficacy and safety	[90]
32	de Oliveira et al., 2025	Rocatinlimab and other anti-OX40 therapies	Improved eczema severity, pruritus, and clinical response rates with acceptable safety.	Supports OX40 inhibition as a promising emerging systemic therapy for moderate-to-severe AD.	[91]
33	Pereyra-Rodriguez et al., 2021	Dupilumab, tralokinumab, lebrikizumab, nemolizumab, baricitinib, abrocitinib, upadacitinib	Upadacitinib and abrocitinib showed the highest EASI-75 and EASI-90 responses.	Supports JAK inhibitors for rapid control of moderate-to-severe AD	[92]
34	Arana et al., 2021	TCIs, TCS	Meta-analysis: Small risk of lymphoma but absolute risk negligible.	Clarifies benefit–risk balance	[93]
35	Chen et al., 2023	Dupilumab, tralokinumab, lebrikizumab, abrocitinib, upadacitinib, baricitinib	JAK inhibitors showed faster and greater short-term EASI improvement than biologics.	Supports JAK inhibitors for rapid symptom control in moderate-to-severe AD	[94]
36	Zhang et al., 2021	Upadacitinib, abrocitinib, baricitinib	Improved EASI-75, EASI-90, and itch outcomes; higher doses increased adverse events.	Supports JAK inhibitor use with the need for safety monitoring	[95]
37	Zheng et al., 2024	Dupilumab, lebrikizumab, tralokinumab, nemolizumab	Improved EASI-75 and pruritus with favorable safety.	Supports personalized biologic selection	[96]

**Table 3:** Comparative effectiveness and evolving strategies for calcineurin inhibitors vs. biologics, JAK inhibitors, and novel formulations.

Feature	CNIs	Biologics (Dupilumab, Tralokinumab)	JAK Inhibitors (Ruxolitinib)	Novel CNI Delivery	References
Site of action	Local, skin-specific inhibition of T-cell activation via calcineurin–NFAT pathway	Systemic (IL-4Rα/IL-13 axis)	Systemic (JAK–STAT signaling)	Skin-specific with enhanced epidermal penetration	[122,123,124]
Onset of action	Days–weeks	Weeks	Rapid (days)	Potentially faster onset due to improved skin penetration compared with conventional CNIs	[125,126,127]
Efficacy (EASI reduction)	Strong efficacy in mild-to-moderate AD, particularly facial and flexural involvement; suitable for long-term maintenance; Moderate but sustained improvement; EASI-50 achieved in ~40–60% with proactive use	High systemic disease control	Rapid itch relief, systemic modulation	Promising efficacy in preclinical and early clinical studies	[128,129]
Safety	Transient burning and pruritus common at initiation; favorable long-term safety profile with minimal systemic absorption	Conjunctivitis, eosinophilia	Infections, thrombosis, lab monitoring	Designed to reduce local irritation while maintaining safety	[130]
Cost and accessibility	Widely available, lower cost	High	High, requires monitoring	Expected to be cost-effective if scaled for clinical use	[131]
Pediatric use	Approved ≥ 2 years	≥6 years	Limited	To be established	[132]
Combination strategies	Proactive maintenance with emollients	Combined with CS in severe AD	Under trial in combinations	CNI + ceramides/barrier agents show synergy	[133]
Future perspective	Global accessibility, safe steroid-sparing	Precision medicine systemic therapy	Effective but with safety trade-offs	Integration into precision dermatology	[134]
IGA response	IGA 0/1 achieved in ~30–40% with continued therapy	-	IGA 0/1 achieved in ~50% by week 8	-	[130]

**Table 4:** Emerging nanocarrier and advanced delivery platforms for topical calcineurin inhibitors (tacrolimus and pimecrolimus) in atopic dermatitis, summarizing carrier types, formulation approaches, key clinical outcomes and the supporting literature evidence.

Sr.	Carrier	Formulation Approach	Drug	Key Outcomes	Reference
1.	Solid lipid nanoparticles (SLN)	SLN-chitosan-coated SLN gel	Tacrolimus	Enhanced skin retention and sustained release; high EE%; reduced irritancy vs. ointment (preclinical/in vitro).	[139]
2.	Thermo-responsive SLNs	Thermosensitive SLN hydrogel	Tacrolimus	Increased dermal penetration and deeper skin distribution; improved retention and tolerability in vivo.	[140]
3.	Nanostructured lipid carriers (NLC)	NLC-based nanogel	Tacrolimus	Improved skin deposition, better aesthetic/handling properties; potential dose-sparing (preclinical).	[141]
4.	Transfersomes (ultra-deformable vesicles)	Transfersomal gel/vesicles	Tacrolimus	Superior transdermal delivery vs. liposomes; improved efficacy in dermatitis models.	[142]
5.	Ethosomes/transethosomes	Ethosomal gel	Tacrolimus	Increased stratum corneum flux and dermal uptake; favorable permeability (preclinical).	[143]
6.	Microemulsion	O/W microemulsion gel	Tacrolimus	Enhanced permeation and steady release; promising early clinical/phase data for topical use.	[144]
7.	Nanoemulsions	Oil-in-water nanoemulsion	Tacrolimus	Increased flux and skin retention; improved tolerability vs. conventional vehicle.	[145]
8.	Liposomes/deformable liposomes	Conventional and elastic liposomes	Tacrolimus	Increased local skin levels; transfersomes often show superior deep penetration.	[144,146]
9.	Polymeric nanoparticles (e.g., chitosan)	Chitosan-coated polymeric NPs/gels	Tacrolimus/Pimecrolimus	Mucoadhesive/positive-charge benefits; enhanced retention and reduced systemic permeation (preclinical).	[147]
10.	Polymeric micelles	PEG/PLGA micelles	Tacrolimus	Solubilization of hydrophobic drug; controlled release and improved dermal delivery in vitro.	[147]
11.	Nanocrystals/nanosuspensions	Nanocrystal suspension/incorporated into gels/patches	Tacrolimus	Increased dissolution rate, enhanced skin uptake; suitable for patch/controlled systems.	[145,146]
12.	Microneedle-assisted delivery	Dissolving/coated microneedles loaded with CNIs	Tacrolimus nanocrystals in MN patch	Bypass SC barrier—rapid intradermal delivery; promising for recalcitrant plaques (preclinical/early translational).	[148]
13.	Cubosomes/GMO-based lipid nanoparticles	Cubosome dispersion/gel	Tacrolimus	High loading and sustained release; promising dermal targeting (preclinical).	[142]
14.	Dendrimers (PAMAM etc.)	Drug–dendrimer complex/gel	Tacrolimus	High drug loading and controlled release; early-stage reports show enhanced skin delivery.	[144,146]
15.	Pro-liposomes/niosomes	Dry precursor-liposome/niosomal dispersion at use	Tacrolimus	Improved storage stability and in situ liposome formation; enhanced dermal targeting (preclinical).	[146,147,148]
16.	Spray-dried SLN powders	Dry SLN powders for reconstitution/patch	Tacrolimus	Improved storage stability, potential for dry-dosage formats.	[149]
17.	Supercritical fluid processed particles	Supercritical CO_2_–derived lipid NPs	Tacrolimus	Solvent-free particle generation with tight size control; equipment-intensive but solvent-safe.	[150]
18.	Combination (co-loaded) NLCs/SLNs	CNI + barrier/antioxidant co-loading	Tacrolimus ± ceramides/antioxidant	Synergistic anti-inflammatory + barrier repair effects; improved clinical indices in preclinical models.	[151]
19.	Pimecrolimus nano-systems	Lipid/polymer vesicles, nanoemulsions, NLCs	Pimecrolimus	Enhanced dermal targeting, high EE%, reduced irritation; suitable for pediatric-sensitive sites (preclinical).	[152]

## Data Availability

No new data were created or analyzed in this study.

## List of Abbreviations

AD	Atopic dermatitis
AAP	American Academy of Pediatrics
APC	Antigen-presenting cell
EASI	Eczema Area and Severity Index
FDA	Food and Drug Administration
IGA	Investigator’s Global Assessment
IL	Interleukin
IMD	Immune-mediated dysfunction
JAK	Janus kinase
MW	Molecular weight
NFAT	Nuclear factor of activated T cells
STAT	Signal transducer and activator of transcription
TCI	Topical calcineurin inhibitor
TCS	Topical corticosteroid
Th	T-helper (cell)
